# Correlations and influencing factors of vitamin A and D levels in maternal and neonatal cord blood among pregnant women

**DOI:** 10.3389/fendo.2026.1878854

**Published:** 2026-07-27

**Authors:** Rui Pu, Su Liu, Yong Liu, Ying Liu, Xuening Li, Jia He

**Affiliations:** 1 Department of Pediatric Intensive Care Unit, The Seventh Affiliated Hospital of Sun Yat-Sen University, Shenzhen, Guangdong, China; 2 Pediatric Hematology Laboratory, Division of Hematology/Oncology, Department of Pediatrics, The Seventh Affiliated Hospital of Sun Yat-Sen University, Shenzhen, Guangdong, China; 3 Department of Pediatrics, The Fourth Affiliated Hospital of China Medical University, Shenyang, Liaoning, China

**Keywords:** maternal, Third trimester of pregnancy, umbilical cord blood, Vit A, vit D

## Abstract

**Background:**

This study aimed to determine the correlation between maternal third-trimester vitamin A(Vit A)and D levels and neonatal cord blood (CB) concentrations, and to identify modifiable maternal factors influencing neonatal status.

**Methods:**

In this cohort of 118 mother-neonate pairs, maternal venous blood levels and CB samples were analyzed. Vit A and 25-hydroxyvitamin D were measured via high-performance liquid chromatography (HPLC) and HPLC coupled with tandem mass spectrometry (HPLC-MS/MS), respectively. Associations were assessed using Pearson correlation and logistic regression.

**Results:**

CB Vit A was significantly lower, while CB Vit D was higher than maternal levels (both *P* < 0.05). Positive mother-neonate correlations were observed for both vitamins (Vit A: r=0.256; Vit D: r=0.697). Better maternal sleep, higher Vit A intake, and frequent vegetable consumption were associated with higher CB Vit A. Maternal physical activity (>1 hour/day) and Vit D supplementation were associated with favorable CB Vit D levels.

**Conclusions:**

Neonatal Vit A and D status is positively correlated with maternal third-trimester levels and is associated with specific, modifiable maternal lifestyle and dietary factors, informing targeted prenatal nutritional strategies.

## 1 Introduction

Vitamin (Vit) A and D are crucial fat-soluble vitamins for human body. They participate in multiple pivotal physiological processes including growth and development, immune regulation, bone metabolism and epithelial tissue repair. Given their highly homologous absorption and metabolic pathways, the two vitamins exert synergistic regulatory effects on fetal intrauterine development, serving as crucial micronutrients for maintaining normal pregnancy and neonatal health (1, 2). Vit A is essential for maintaining visual acuity, epithelial integrity, normal growth and development, robust immune responses, and the prevention of anemia (3). Vit A plays a fundamental role in developing the neonatal nervous, respiratory, and physical systems and influences neonatal immune function and survival rates (4–6). Maternal Vit A imbalance or deficiency during pregnancy markedly increases the risks of adverse outcomes such as fetal intrauterine growth restriction, low neonatal immunity and respiratory diseases, which further exert long-term impacts on the growth development and survival rate of newborns (7). Maternal-to-neonatal Vit A transfer actively occurs via the placenta (8). Thus, clarifying the maternal-neonatal Vit A correlation in the third trimester provides critical clinical guidance for improving neonatal Vit A nutritional status. Previous studies indicated that children’s Vit A levels can be associated with age, sex, family economic income, parental education, and eating habits (9, 10). Most existing studies focus on nutritional factors in childhood. Few investigations systematically explore controllable prenatal factors including dietary patterns, lifestyle, Vit A intake and supplementation compliance. Additionally, the correlation between maternal multidimensional exposure factors and neonatal Vit A reserves remains poorly verified, hindering the formulation of targeted prenatal nutritional intervention strategies.

Vit D maintains the dynamic balance of calcium and phosphorus in the body, supports skeletal health, and potentially contributes to muscle function and immune responses. Maternal Vit D insufficiency in the mid to late stages of pregnancy can heighten the risk of unfavorable pregnancy outcomes and hinder fetal growth (11–13). Vit D primarily crosses the placenta as 25(OH)D, which fetal kidneys subsequently convert to its active form, 1,25(OH)2D (14). Consequently, CB 25(OH)D is critical for neonatal Vit D reserves (15). Numerous domestic and international studies have explored the correlation of Vit D between mothers and cord blood, basically clarifying its placental transport patterns and influencing factors. However, most studies only analyze Vit D separately, without simultaneous comparison of the correlation differences between Vit A and D in the same cohort and fail to deeply explore individualized influencing factors such as regional dietary habits and daily routines.

Most existing studies separately investigate maternal and neonatal Vit A and D nutrition, ignoring their homologous fat-soluble properties and synergistic effects on fetal development, leading to research limitations. Relevant domestic investigations are mainly conducted in southern, coastal and central China, while no specific maternal-infant cohort studies have been carried out in Northeast China. Shenyang has distinctive local dietary and lifestyle habits. The nutritional profiles, placental transport mechanisms and influencing factors of the two vitamins among local mothers and infants remain unclear, lacking evidence for localized nutritional intervention.

Based on the above research status, this study enrolled late pregnant women and their newborns in Shenyang. Serum and cord blood concentrations of Vit A and Vit D were determined to explore their placental transfer correlation. Multidimensional factors including socioeconomic status, diet, lifestyle and nutritional supplementation were analyzed to screen independent influencing factors of neonatal vitamin reserves. The findings can provide evidence for local prenatal nutritional intervention and improve maternal and infant nutritional health.

## 2 Materials and methods

### 2.1 Study population

Data were collected from mother-infant pairs who delivered at the Fourth Affiliated Hospital of China Medical University from September 2019 to February 2020. Eligibility criteria included pregnant women aged at least 20 years, gestational periods ranging from 37 to 42 weeks, long-term residence in Shenyang, and the absence of previous cardiovascular, endocrine, urinary, metabolic, autoimmune, or neuromuscular disorders. Neonates who experienced birth asphyxia, congenital organ malformations, chromosomal anomalies, inherited metabolic conditions, infection symptoms (e.g., fever), or those conceived through assisted reproductive methods were excluded (16). The Ethics Committee approved the study(Ethics: EC-2019-KS-027), and informed consent was formally obtained from all participants.

### 2.2 Data collection

Maternal participants completed two self-administered questionnaires upon admission: a Pregnancy General Condition Questionnaire and a Pregnancy Food Frequency Questionnaire (FFQ). The general questionnaire collected maternal characteristics, lifestyle factors, pregnancy-related conditions, and nutritional supplement intake. Neonatal characteristics were obtained from birth records. The FFQ assessed intake frequencies of various food categories. Intake frequency categories included none, once/month, once/week, 2–3 times/week, 4–5 times/week, once/day, 2–3 times/day, or >3 times/day.

### 2.3 Sample collection and assays

Maternal blood was sampled one week before delivery at full-term gestation (37–42 weeks gestation) during late third-trimester prenatal visits. Umbilical cord blood was collected right after delivery to form matched mother-neonate pairs. Serum retinol (Vit A) and total 25-hydroxyvitamin D [total 25(OH)D] concentrations were quantified via high-performance liquid chromatography-tandem mass spectrometry (HPLC-MS/MS). The testing credentials of the laboratory have been previously described (17). Identical analytical procedures were conducted for both neonatal CB and maternal venous blood(MVB).

### 2.4 Diagnostic criteria

#### 2.4.1 Mothers

Vit A status was considered sufficient at levels of 0.3 mg/L or higher; marginal Vit A deficiency (MVAD) was defined as levels from 0.2 mg/L up to but not including 0.3 mg/L; and deficiency (VAD) was identified as concentrations below 0.2 mg/L (18). For Vit D, adequate status was established as total 25(OH)D concentrations above 30 ng/mL, insufficiency ranged between 20 ng/mL to 30 ng/mL, and deficiency was categorized as levels at or below 20 ng/mL (19).

#### 2.4.2 Newborns

Alternative classification standards used were Vit A sufficiency at levels equal to or exceeding 0.2 mg/L, MVAD between 0.1 mg/L to under 0.2 mg/L, and VAD below 0.1 mg/L (18). Vit D sufficiency was considered above 20 ng/mL, insufficiency between 12 ng/mL to 20 ng/mL, and deficiency below 12 ng/mL (20).

### 2.5 Statistical analysis

Continuous data following a normal distribution were reported as mean ± standard deviation (SD), while categorical variables were represented as counts and percentages [n (%)]. Pearson correlation and paired-sample t-tests evaluated the relationships between maternal third-trimester and neonatal umbilical CB Vit A and Vit D concentrations. Chi-square tests assessed associations between influencing factors, stratified by median dietary frequency during pregnancy. A two-step approach was applied for variable selection in multivariable binary logistic regression: variables significant at (*P* < 0.05) on univariate testing entered the candidate set, while maternal age and gestational weight gain were forced into the final adjusted model owing to their well-documented clinical confounding effects in perinatal nutrition studies (21) Statistical analyses were conducted using SPSS software version 23.0, with significance defined at *P* < 0.05.

## 3 Results

### 3.1 Vit A and D Levels in CB and third-trimester MVB

**Figure 1:**
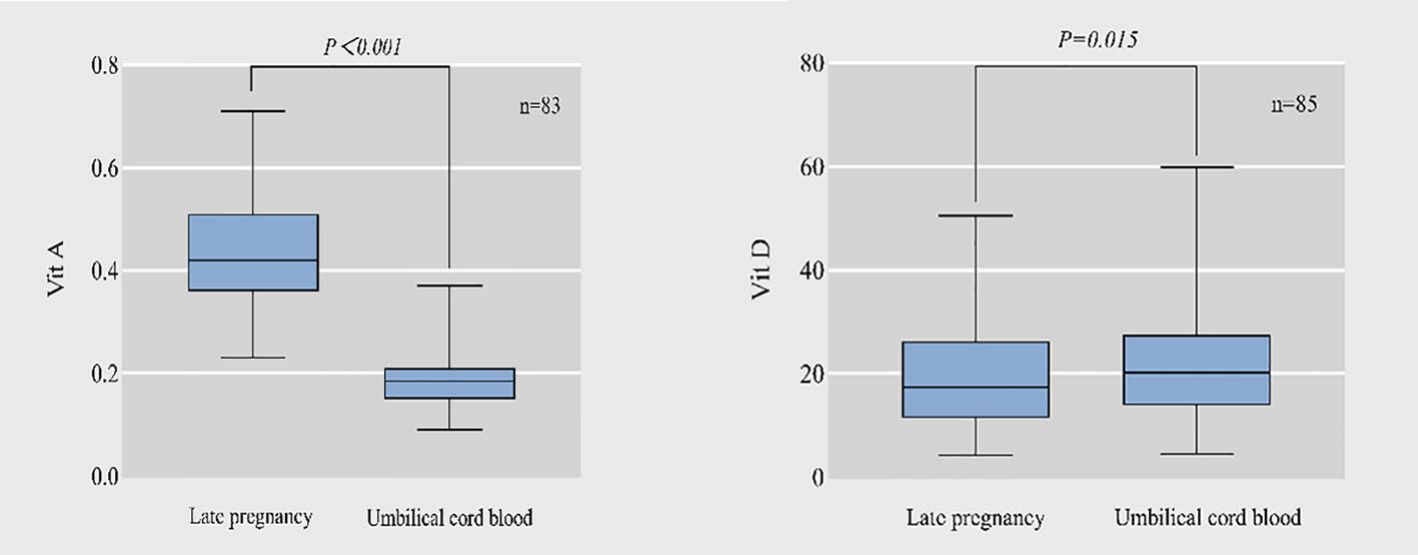
Distribution of Vit A and D levels in third-trimester MVB and CB.

A total of 118 mother-neonate paired participants were initially enrolled in this study. Due to loss to follow-up during sample collection, serum Vit A data were missing from 35 maternal specimens, and serum Vit D data were missing from 33 maternal specimens. Consequently, the final valid paired datasets for maternal-cord blood Vit A and Vit D analyses comprised 83 pairs and 85 pairs, respectively. Sensitivity analyses were performed on these complete paired datasets, which confirmed that all core findings were robust. Neonatal umbilical CB samples from all 118 pairs were analyzed for Vit A and D levels. **Table 1** presents comprehensive details on maternal and neonatal vitamin concentrations. The average CB Vit A level was 0.19 ± 0.05 mg/L, with rates of MVAD and VAD at 58.47% and 3.39%, respectively. In comparison, MVB Vit A averaged 0.44 ± 0.11 mg/L, with an MVAD rate of 7.23% and no cases of VAD. CB Vit A concentrations were significantly lower (*P* < 0.05, **Figure 1**). CB Vit D concentrations averaged 21.51 ± 8.53 ng/mL, exhibiting deficiency, insufficiency, and sufficiency rates of 8.47%, 41.53%, and 50.00%, respectively. In MVB, third-trimester Vit D levels averaged 19.67 ± 10.03 ng/mL, with corresponding rates of 56.47% deficiency, 27.06% insufficiency, and 16.47% sufficiency. CB Vit D concentrations were notably higher than MVB (*P* < 0.05, **Figure 1**).

**Table 1:** Distribution of Vit A and D levels in third-trimester MVB and CB.

Group	Classification	N	%
CB Vit A Level	VAD	4	3.39
	MVAD	69	58.47
	sufficient	45	38.14
CB Vit D Level	deficient	10	8.47
	insufficient	49	41.53
	sufficient	59	50.00
Third-Trimester MVB Vit A	VAD	0	0
	MVAD	6	7.23
	sufficient	77	92.77
Third-Trimester MVB Vit D	deficient	48	56.47
	insufficient	23	27.06
	sufficient	14	16.47

### 3.2 Correlation analysis of Vit A and D levels in CB and third-trimester MVB

**Figure 2:**
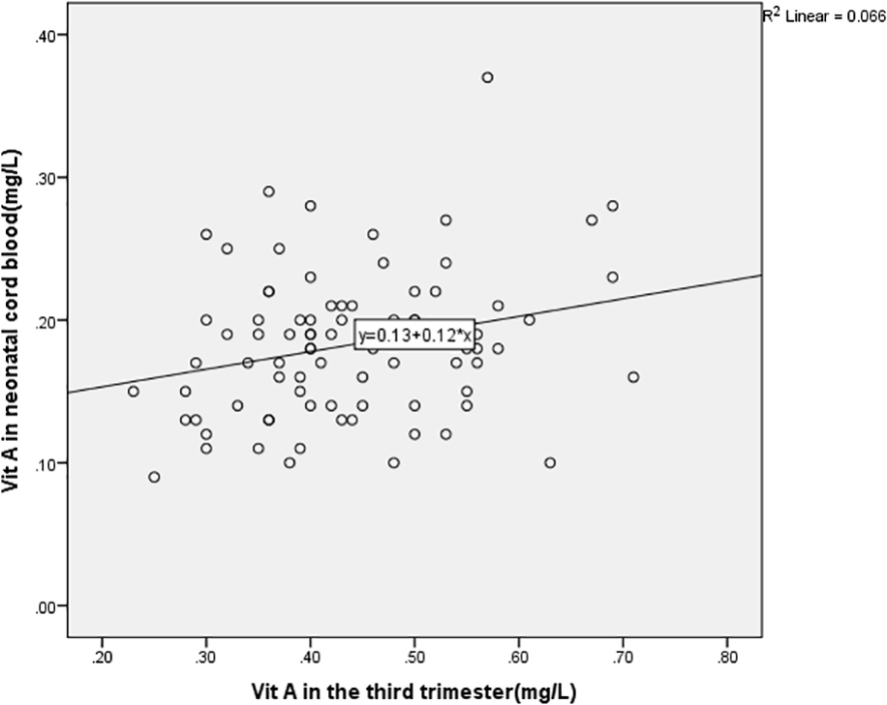
Correlation of Vit A levels in CB and third-trimester MVB.

**Figure 3:**
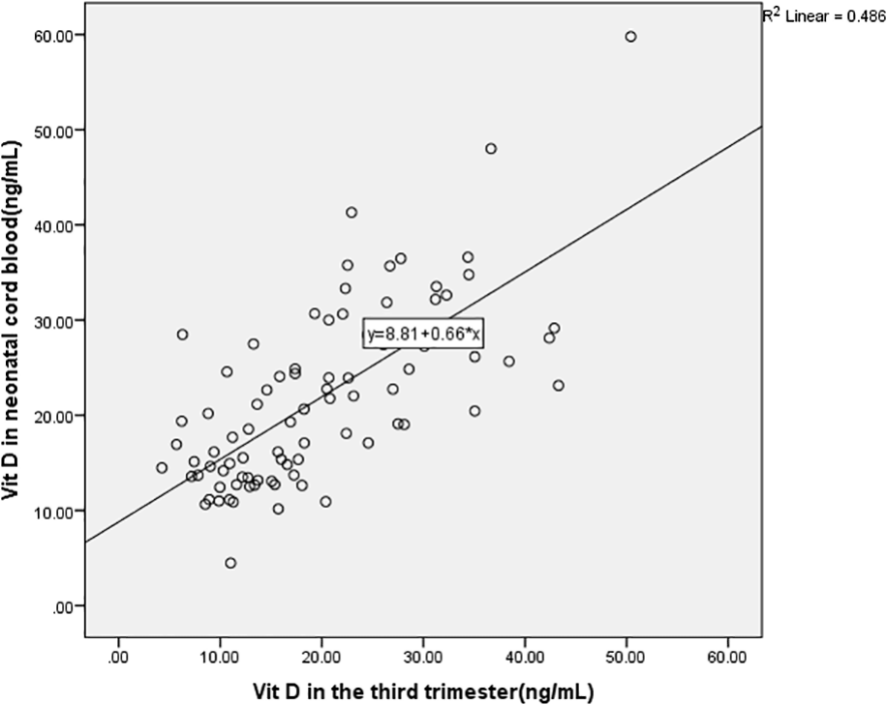
Correlation of Vit D levels in CB and third-trimester MVB.

Pearson correlation analyses demonstrated a significant positive correlation between maternal venous and neonatal CB Vit A levels (r=0.256, *P* = 0.019, **Figure 2**). Additionally, a stronger positive correlation was identified for Vit D levels (r=0.697, *P* < 0.001, **Figure 3**).

### 3.3 Univariate Analysis of Factors Influencing CB Vit A and D Levels

#### 3.3.1 Effect of third-trimester maternal general condition on CB Vit A levels

Neonatal CB Vit A was grouped into adequate levels and marginal deficiency or deficiency. Maternal influences on neonatal Vit A status were subsequently assessed. Chi-square tests demonstrated significant correlations between neonatal Vit A status and maternal outdoor activity, sleep quality during pregnancy, and supplementation with Vit A (all *P* < 0.05), while other maternal characteristics showed no significant differences (all *P*>0.05, **Table 2**).

**Table 2:** Univariate analysis of CB Vit A levels.

Variable	Group	Vit A levels (mg/L)		χ2	P
		<0.2	≥0.2		
Age (years)	≤30	51 (65.4)	27 (34.6)	1.209	0.272
	>30	22 (55.0)	18 (45.0)		
Ethnicity	Han	63 (63.6)	36 (36.4)	0.818	0.366
	Others	10 (52.6)	9 (47.4)		
Educational level	High school or below	21 (63.6)	12 (36.4)	0.061	0.805
	College or higher	52 (61.2)	33 (38.8)		
Place of residence	Urban	67 (61.5)	42 (38.5)	0.095	1.000
	Rural	6 (66.7)	3 (33.3)		
Income	Comfortable	60 (59.4)	41 (40.6)	1.796	0.180
	Moderate	13 (76.5)	4 (23.5)		
Gestational weight gain*	Insufficient	7 (53.8)	6 (46.2)	0.735	0.692
	Suitable	24 (66.7)	12 (33.3)		
	Excessive	42 (60.9)	27 (39.1)		
Outdoor time	≤1 h/day	62 (66.7)	31 (33.3)	4.291	**0.038**
	>1 h/day	11 (44.0)	14 (56.0)		
History of URI	Yes	40 (62.5)	24 (37.5)	0.024	0.877
	No	33 (61.1)	21 (38.9)		
Gestational complications	Yes	24 (61.5)	15 (38.5)	0.003	0.959
	No	49 (62.0)	30 (38.0)		
Gestational Sleep quality	Good	45 (54.9)	37 (45.1)	5.561	**0.018**
	Fair	28 (77.8)	8 (22.2)		
DHA supplementation	Yes	12 (52.2)	11 (47.8)	1.137	0.286
	No	61 (64.2)	34 (35.8)		
Iron supplementation	Yes	37 (62.7)	22 (37.3)	0.036	0.850
	No	36 (61.0)	23 (39.0)		
Vit A supplementation	Yes	30 (49.2)	31 (50.8)	8.612	**0.003**
	No	43 (75.4)	14 (24.6)		
Preconceptional Passive Smoking Exposure	Yes	34 (64.2)	19 (35.8)	0.213	0.644
	No	39 (60.0)	26 (40.0)		
Postpartum Passive Smoking Exposure	Yes	26 (63.4)	15 (36.6)	0.064	0.800
	No	47 (61.0)	30 (39.0)		
Pet ownership	Yes	11 (78.6)	3 (21.4)	1.879	0.170
	No	62 (59.6)	42 (40.4)		
Gestational Employment	Yes	50 (67.6)	24 (32.4)	2.736	0.098
	No	23 (52.3)	21 (47.7)		

Bold font indicates *P* < 0.05. *Recommended weight gain during pregnancy (22): 12.5-18.0 kg (BMI <18.5 kg/m^2^), 11.5-16.0 kg (BMI 18.5-25.0 kg/m^2^), 7.0-11.5 kg (BMI 25.0-30.0 kg/m^2^), and 5.0-9.0 kg (BMI ≥30.0 kg/m^2^).

#### 3.3.2 Effect of Third-Trimester Maternal General Condition on CB Vit D Levels

CB Vit D concentrations were categorized into normal/insufficient and deficient groups. Chi-square tests indicated significant differences in maternal education, outdoor activity duration, and Vit D supplementation between the two groups (all *P* < 0.05). No statistically significant differences were detected for other examined variables (all *P*>0.05, **Table 3**).

**Table 3:** Univariate analysis of CB Vit D levels.

Variable	Group	Vit D levels (ng/mL)		χ2	P
		<20	≥20		
Age (years)	≤30	43 (55.1)	35 (44.9)	2.421	0.120
	>30	16 (40.0)	24 (60.0)		
Ethnicity	Han	49 (49.5)	50 (50.5)	0.063	0.802
	Others	10 (52.6)	9 (47.4)		
Educational level	High school or below	22 (66.7)	11 (33.3)	5.090	**0.024**
	College or higher	37 (43.5)	48 (56.5)		
Place of residence	Urban	56 (51.4)	53 (48.6)	1.083	0.490
	Rural	3 (33.3)	6 (66.7)		
Income	Comfortable	52 (51.5)	49 (48.5)	0.619	0.432
	Moderate	7 (41.2)	10 (58.8)		
Gestational weight gain	Insufficient	6 (46.2)	7 (53.8)	0.884	0.643
	Suitable	16 (44.4)	20 (55.6)		
	Excessive	37 (53.6)	32 (46.4)		
Outdoor time	≤1 h/day	56 (60.2)	37 (39.8)	18.322	**<0.001**
	>1 h/day	3 (12.0)	22 (88.0)		
History of URI	Yes	36 (56.3)	28 (43.7)	2.185	0.139
	No	23 (42.6)	31 (57.4)		
Gestational complications	Yes	19 (48.7)	20 (51.3)	0.038	0.845
	No	40 (50.6)	39 (49.4)		
Gestational Sleep quality	Good	40 (48.8)	42 (51.2)	0.160	0.689
	Fair	19 (52.8)	17 (47.2)		
DHA supplementation	Yes	10 (43.5)	13 (56.5)	0.486	0.486
	No	49 (51.6)	46 (48.4)		
Iron supplementation	Yes	27 (45.8)	32 (54.2)	0.847	0.357
	No	32 (54.2)	27 (45.8)		
Vit D supplementation	Yes	37 (41.6)	52 (58.4)	10.287	**0.001**
	No	22 (75.9)	7 (24.1)		
Calcium supplementation	Yes	40 (47.6)	44 (52.4)	0.661	0.416
	No	19 (55.9)	15 (44.1)		
Preconceptional Passive Smoking Exposure	Yes	26 (49.1)	27 (50.9)	0.034	0.853
	No	33 (50.8)	32 (49.2)		
Postpartum Passive Smoking Exposure	Yes	20 (48.8)	21 (51.2)	0.037	0.847
	No	39 (50.6)	38 (49.4)		
Pet ownership	Yes	7 (50.0)	7 (50.0)	<0.001	1.000
	No	52 (50.0)	52 (50.0)		
Gestational Employment	Yes	36 (48.6)	38 (51.4)	0.145	0.703
	No	23 (52.3)	21 (47.7)		

Bold font indicates *P* < 0.05.

#### 3.3.3 Effects of gestational dietary frequency on CB Vit A levels

Neonatal CB Vit A concentrations were classified into normal or marginal/deficient categories. Associations between maternal dietary intake during pregnancy and neonatal CB Vit A status were assessed. Chi-square tests revealed significant differences between groups in the intake frequencies of animal liver, eggs, dairy products, soy products, vegetables, and fruits (all *P* < 0.05). Consumption frequencies of other food items showed no significant differences between the two groups (all *P*>0.05, **Table 4**).

**Table 4:** Effects of gestational dietary frequency on CB Vit A levels.

Food type	Frequency	Vit A level (mg/L)		χ2	P
		<0.2	≥0.2		
Staple foods	≤2 times/day	30 (73.2)	11 (26.8)	3.404	0.065
	>2 times/day	43 (55.8)	34 (44.2)		
Coarse grains	<1 time/day	66 (63.5)	38 (36.5)	0.948	0.330
	≥1 time/day	7 (50.0)	7 (50.0)		
Lean meat	<1 time/day	46 (66.7)	23 (33.3)	1.624	0.202
	≥1 time/day	27 (55.1)	22 (44.9)		
Animal liver	≤1 time/week	50 (69.4)	22 (30.6)	4.498	0.034
	>1 time/week	23 (50.0)	23 (50.0)		
Eggs	<1 time/day	48 (70.6)	20 (29.4)	5.177	0.023
	≥1 time/day	25 (50.0)	25 (50.0)		
Dairy products	≤1 time/day	47 (70.1)	20 (29.9)	4.520	0.034
	>1 time/day	26 (51.0)	25 (49.0)		
Soy products	≤3 times/week	55 (68.8)	25 (31.3)	4.992	0.025
	>3 times/week	18 (47.4)	20 (52.6)		
Seafood	≤1 time/week	53 (62.4)	32 (37.6)	0.031	0.861
	>1 time/week	20 (60.6)	13 (39.4)		
Vegetables	≤1 time/day	42 (77.8)	12 (22.2)	10.687	0.001
	>1 time/day	31 (48.4)	33 (51.6)		
Fruits	≤1 time/day	43 (71.7)	17 (28.3)	4.971	0.026
	>1 time/day	30 (51.7)	28 (48.3)		
Cooked meat sausage	≤1 time/month	37 (61.7)	23 (38.3)	0.002	0.964
	>1 time/month	36 (62.1)	22 (37.9)		
Canned food	No intake	59 (60.8)	38 (39.2)	0.250	0.617
	≥1 time/month	14 (66.7)	7 (33.3)		
Barbecue	No intake	37 (61.7)	23 (38.3)	0.002	0.964
	≥1 time/month	36 (62.1)	22 (37.9)		
Fried food	No intake	25 (67.6)	12 (32.4)	0.743	0.389
	≥1 time/month	48 (59.3)	33 (40.7)		
Nuts	<4 times/month	45 (66.2)	23 (33.8)	1.265	0.261
	≥4 times/month	28 (56.0)	22 (44.0)		
Sweet food	≤1 time/month	30 (58.8)	21 (41.2)	0.352	0.553
	>1 time/month	43 (64.2)	24 (35.8)		
Puffed food	<1 time/week	56 (58.9)	39 (41.1)	1.758	0.185
	≥1 time/week	17 (73.9)	6 (26.1)		
Beverages	No intake	40 (59.7)	27 (40.3)	0.307	0.579
	≥1 time/month	33 (64.7)	18 (35.3)		

Bold font indicates *P* < 0.05.

### 3.4 Multivariate analysis of factors influencing CB Vit A and Vit D levels

#### 3.4.1 Multifactorial analysis of CB Vit A levels

To further explore the determinants influencing neonatal Vit A levels, binary logistic regression analyses were performed. Multivariate binary logistic regression was conducted by incorporating variables with *P* < 0.05 in univariate analysis, as well as pre-specified clinically relevant covariates (age and maternal gestational weight gain) to control for confounding bias. According to the multivariate results, adequate sleep quality during pregnancy (OR = 0.331, 95% CI: 0.120–0.915), supplementation with Vit A (OR = 0.381, 95% CI: 0.147–0.989), and frequent daily vegetable consumption (>1 time/day) (OR = 0.334, 95% CI: 0.124–0.902) were associated with higher CB Vit A status (all *P* < 0.05). Other analyzed variables did not exhibit significant correlations (all *P*>0.05, **Tables 5**, **6**).

**Table 5:** Binary logistic regression analysis of CB Vit A level-related factors.

Influencing factor		B	SE	Wald	P	OR (95% CI)
Outdoor time	≤1 h/day	0^a^				
	>1 h/day	-0.723	0.584	1.533	0.216	0.486 (0.155-1.524)
Gestational Sleep quality	Good	0^a^				
	Fair	-1.106	0.519	4.544	**0.033**	0.331 (0.120-0.915)
Vit A supplementation	No	0^a^				
	Yes	-0.966	0.487	3.930	**0.047**	0.381 (0.147-0.989)
Animal liver	<1 time/week	0^a^				
	≥1 time/week	-0.605	0.493	1.504	0.220	0.546 (0.208-1.436)
Eggs	<1 time/day	0^a^				
	≥1 time/day	-0.421	0.494	0.724	0.395	0.657 (0.249-1.730)
Dairy products	<1 time/day	0^a^				
	≥1 time/day	-0.177	0.480	0.136	0.713	0.838 (0.327-2.147)
Soy products	≤3 times/week	0^a^				
	>3 times/week	-0.613	0.474	1.675	0.196	0.542 (0.214-1.371)
Vegetables	≤1 time/day	0^a^				
	>1 time/day	-1.096	0.507	4.685	**0.030**	0.334 (0.124-0.902)
Fruits	≤1 time/day	0^a^				
	>1 time/day	-0.214	0.495	0.187	0.665	0.807 (0.306-2.129)

^a^reference group; bold = *P* < 0.05; CB Vit A level was used as the dependent variable (1: <0.2 mg/L, 0: ≥0.2 mg/L).

**Table 6:** Adjusted binary logistic regression for predictors of umbilical cord blood Vit A level.

Influencing factor		B	SE	Wald	P	OR (95% CI)
Outdoor time	≤1 h/day	0^a^				
	>1 h/day	0.604	0.602	1.006	0.316	1.829 (0.562-5.950)
Gestational Sleep quality	Good	0^a^				
	Fair	1.082	0.531	4.148	**0.042**	2.950 (1.042-8.357)
Vit A supplementation	No	0^a^				
	Yes	-1.042	0.499	4.366	**0.037**	0.353 (0.133-0.937)
Animal liver	<1 time/week	0^a^				
	≥1 time/week	-0.580	0.503	1.331	0.249	0.560 (0.209-1.500)
Eggs	<1 time/day	0^a^				
	≥1 time/day	-0.458	0.502	0.832	0.362	0.633 (0.236-1.693)
Dairy products	<1 time/day	0^a^				
	≥1 time/day	-0.117	0.488	0.057	0.811	0.889 (0.341-2.317)
Soy products	≤3 times/week	0^a^				
	>3 times/week	-0.578	0.476	1.474	0.225	0.561 (0.221-1.427)
Vegetables	≤1 time/day	0^a^				
	>1 time/day	-1.035	0.512	4.075	**0.044**	0.355 (0.130-0.970)
Fruits	≤1 time/day	0^a^				
	>1 time/day	0.298	0.517	0.331	0.565	1.347 (0.489-3.710)
Gestational weight gain	Inappropriate	0^a^				
	Suitable	-0.109	0.510	0.045	0.831	0.897 (0.330-2.439)
Age (years)	≤30	0^a^				
	>30	-0.303	0.493	0.378	0.539	0.738 (0.281-1.942)

^a^reference group; bold = *P* < 0.05; CB Vit A level was used as the dependent variable (1: <0.2 mg/L, 0: ≥0.2 mg/L).

#### 3.4.2 Multivariate analysis of CB Vit D levels

Likewise, binary logistic regression was conducted to explore determinants influencing neonatal CB Vit D status. Multivariate binary logistic regression was conducted by incorporating variables with *P* < 0.05 in univariate analysis, as well as pre-specified clinically relevant covariates (age and maternal gestational weight gain) to control for confounding bias Results indicated that maternal outdoor exposure exceeding 1 hour per day (OR = 0.103, 95% CI: 0.028–0.379) and Vit D supplementation during pregnancy (OR = 0.332, 95% CI: 0.118–0.933) were significantly associated with lower odds of neonatal CB Vit D deficiency (both *P* < 0.05), whereas other potential factors were not statistically significant (all *P*>0.05, **Tables 7**, **8**).

**Table 7:** Binary logistic regression analysis of CB Vit D level-related factors.

Influencing factor		B	SE	Wald	P	OR (95% CI)
Educational level	High school or below	0^a^				
	College or higher	-0.874	0.498	3.078	0.079	0.417 (0.157-1.108)
Outdoor time	≤1 h/day	0^a^				
	>1 h/day	-2.276	0.666	11.675	**0.001**	0.103 (0.028-0.379)
Vit D supplementation	No	0^a^				
	Yes	-1.103	0.528	4.372	**0.037**	0.332 (0.118-0.933)

^a^reference group; bold = *P* < 0.05; CB Vit D level was used as the dependent variable (1: <20 ng/mL, 0: ≥20 ng/mL).

**Table 8:** Adjusted binary logistic regression for predictors of umbilical cord blood Vit D level.

Influencing factor		B	SE	Wald	P	OR (95% CI)
Educational level	High school or below	0^a^				
	College or higher	-0.896	0.487	3.394	0.065	0.408 (0.157-1.059)
Outdoor time	≤1 h/day	0^a^				
	>1 h/day	-1.570	0.573	7.507	**0.006**	0.208 (0.068-0.640)
Vit D supplementation	No	0^a^				
	Yes	-1.107	0.514	4.638	**0.031**	0.331 (0.121-0.905)
Age (years)	≤30	0^a^				
	>30	-0.375	0.440	0.727	0.394	0.687 (0.290-1.628)
Gestational weight gain	Inappropriate	0^a^				
	Suitable	0.350	0.451	0.603	0.437	1.419 (0.586-3.436)

^a^reference group; bold = *P* < 0.05; CB Vit D level was used as the dependent variable (1: <20 ng/mL, 0: ≥20 ng/mL).

## 4 Discussion

This study analyzed the correlations of Vit A and Vit D levels between maternal venous blood in the third trimester of pregnancy and neonatal serum and identified the relevant factors affecting the vitamin nutritional status of neonates. It aims to provide clinical evidence for nutritional intervention during pregnancy and the improvement of vitamin reserves in newborns.

A notable positive correlation existed between MVB Vit A concentrations in the third trimester and neonatal CB levels (r=0.256, P = 0.019), which was consistent with the findings of previous studies (23). As fat-soluble vitamins can be transported across the placenta via specific transporters (24), this physiological mechanism explains the correlation of Vit A levels between mothers and their offspring. Compared with the moderate correlation coefficients reported in prior literature, the correlation magnitude observed in the present study was slightly different. This suggests that, in the study population, in addition to maternal Vit A intake, placental transport efficiency and fetal metabolic characteristics are also important factors contributing to individual variations in neonatal Vit A status.

Notably, maternal and neonatal Vit D concentrations exhibited a stronger positive correlation (r=0.697, *P* < 0.001) compared to Vit A. This finding aligns with previously published data from Wierzejska et al. (25). Such disparity in correlation strength may be attributable to distinct transport mechanisms across the placenta. Specifically, Vit D, predominantly in its 25(OH)D form, traverses the placental barrier mainly via passive diffusion (26), which is considerably more effective than the actively regulated transportation mechanism of Vit A. In this study population, the overall concentration of Vit D in neonatal umbilical cord blood was relatively high, with an abnormal detection rate lower than that in maternal blood. Combined with findings from previous studies (27, 28), these results suggest that most pregnant women in this cohort had Vit D insufficiency to varying degrees. The placenta may enhance Vit D supply to the fetus through compensatory transport, which represents a distinctive characteristic of the present study. These findings supplement field data regarding the patterns of maternal-fetal Vit D transfer under conditions of maternal Vit D insufficiency.

With regard to neonatal Vit A status, good gestational sleep quality, prenatal Vit A supplementation, and frequent vegetable intake (>1 time/day) were associated with desirable CB Vit A levels. Vegetables are rich in beta-carotene, a precursor of Vit A, while prenatal supplements can directly increase maternal Vit A reserves. The beneficial effects of these two factors on elevating neonatal Vit A levels have been validated in previous studies (29, 30). Notably, this is the first study to observe a significant correlation between sleep quality and Vit A concentrations in umbilical cord blood among pregnant women in this region. Evidence from NHANES data indicates that short sleep duration is associated with inadequate Vit A intake (31). Considering the regulatory effect of circadian rhythms on nutritional metabolism, we speculate that sleep disturbances may indirectly impair the absorption and utilization of Vit A by disrupting dietary behaviors and interfering with the metabolism of nutrients in the body. Conversely, regular and high-quality sleep can maintain normal metabolic rhythms, thereby ensuring bodily Vit A reserves and its transplacental transport.

Regarding neonatal CB Vit D concentrations, maternal outdoor activities exceeding one hour daily and Vit D supplementation were significantly associated with adequate CB Vit D levels. Increased outdoor exposure facilitates skin synthesis of cholecalciferol (Vit D3) via ultraviolet B (UVB) radiation, representing the predominant Vit D source in humans (32). Combined with exogenous supplementation, these measures effectively raise maternal serum 25(OH)D concentrations. Relying on efficient passive diffusion across the placenta, adequate fetal Vit D supply can be secured, and such findings were further validated in the present cohort. In addition, maternal body mass index (BMI) acts as an important confounding factor influencing Vit D status during gestation. Previous evidence has demonstrated that adipose tissue extensively sequesters Vit D, whereby obese individuals frequently exhibit reduced serum 25(OH)D concentrations and diminished bioavailability of circulating Vit D (33). BMI data of all enrolled pregnant women were collected and analyzed in this study. Our research results did not find a correlation between the increase in the mother’s BMI and the increased risk of the mother’s Vit D deficiency. This might be due to the small sample size of our study.

## 5 Strengths and limitations

This study provides an integrated analysis of maternal-neonatal nutritional status by simultaneously investigating the correlations for vitamins A and D and identifying modifiable maternal determinants. Its primary strength lies in the dual-focus design, which employed precise, gold-standard analytical methods (HPLC and HPLC-MS/MS) to quantify paired maternal and cord blood concentrations of both critical fat-soluble vitamins within a prospective cohort. Beyond confirming significant positive correlations—notably a strong association for 25(OH)D (r=0.697)—the research innovatively identified specific, actionable factors associated with optimal cord blood vitamin status, including maternal sleep quality, dietary habits, physical activity, and supplementation patterns. These findings bridge biochemical assessment with practical prenatal lifestyle counseling, offering a holistic evidence base for interventions aimed at optimizing neonatal vitamin reserves.

However, several limitations must be acknowledged. First, the single-center design and modest sample size may constrain the generalizability of the findings to broader populations with different demographic or socioeconomic characteristics. Second, maternal vitamin levels were measured only at a single time point in the third trimester; the lack of serial measurements throughout gestation precludes an understanding of how dynamic changes in maternal status influence cord blood concentrations. In addition, data related to sleep, dietary intake and outdoor activities were obtained via self-report by participants, which inevitably introduced recall bias. Third, the absence of long-term follow-up data means the clinical and developmental significance of the observed cord blood vitamin levels on infant health outcomes remains unknown. Future studies should therefore aim for larger, multi-center cohorts with longitudinal maternal assessments and extended postnatal follow-up to establish the long-term implications of neonatal Vit A and D status.

## 6 Conclusions

In conclusion, this study confirms positive correlations between maternal third-trimester and neonatal CB Vit A and D levels, with a stronger correlation observed for Vit D. Good maternal sleep quality, Vit A supplementation, and frequent vegetable intake were associated with favorable CB Vit A levels, whereas adequate outdoor activity and Vit D supplementation benefited CB Vit D levels. These findings carry important clinical implications: targeted interventions during pregnancy, including Vit A and D supplementation, increased vegetable consumption, adequate outdoor exposure, and improved sleep quality, should be implemented to optimize maternal vitamin status, enhance neonatal vitamin reserves, and promote healthy fetal and neonatal development.

## Correction note

This article has been corrected with minor changes. These changes do not impact the scientific content of the article.

## Data Availability

The raw data supporting the conclusions of this article will be made available by the authors, without undue reservation.
